# Pathophysiological Implication of Pattern Recognition Receptors in Fetal Membranes Rupture: RAGE and NLRP Inflammasome

**DOI:** 10.3390/biomedicines9091123

**Published:** 2021-08-31

**Authors:** Helena Choltus, Marilyne Lavergne, Coraline De Sousa Do Outeiro, Karen Coste, Corinne Belville, Loïc Blanchon, Vincent Sapin

**Affiliations:** 1 CNRS, INSERM, GReD, Université Clermont Auvergne, 63000 Clermont-Ferrand, France; helena.choltus@uca.fr (H.C.); lavergnemarilyne@gmail.com (M.L.); coralinedesousa.do@gmail.com (C.D.S.D.O.); kcoste@chu-clermontferrand.fr (K.C.); corinne.belville@uca.fr (C.B.); loic.blanchon@uca.fr (L.B.); 2 CHU de Clermont-Ferrand, Biochemistry and Molecular Genetic Department, 63000 Clermont-Ferrand, France

**Keywords:** fetal membranes, inflammation, PRR, RAGE, inflammasome, NLRP

## Abstract

Preterm prelabor ruptures of fetal membranes (pPROM) are a pregnancy complication responsible for 30% of all preterm births. This pathology currently appears more as a consequence of early and uncontrolled process runaway activation, which is usually implicated in the physiologic rupture at term: inflammation. This phenomenon can be septic but also sterile. In this latter case, the inflammation depends on some specific molecules called “alarmins” or “damage-associated molecular patterns” (DAMPs) that are recognized by pattern recognition receptors (PRRs), leading to a microbial-free inflammatory response. Recent data clarify how this activation works and which receptor translates this inflammatory signaling into fetal membranes (FM) to manage a successful rupture after 37 weeks of gestation. In this context, this review focused on two PRRs: the receptor for advanced glycation end-products (RAGE) and the NLRP7 inflammasome.

## 1. Fetal Membranes: A Human Barrier Essential to Childbirth

During pregnancy, the fetus must be in a peaceful and homeostatic environment, hence being protected against external aggression so that it can grow. Fetal membranes (FM), an adjustable biological barrier, are essential to this protection.

### 1.1. Structure of Fetal Membranes

The FM, such as the placenta, are extra-embryonic transient tissues expelled during delivery. The placenta is located on the surface of the uterus wall, while the FMs are attached to the placenta and cover the inner wall of the amniotic cavity containing the fetus and amniotic fluid (AF). With a surface area of around 1500 cm^2^ and a thickness between 200 and 300 µm at term, FM interact with the placenta and maternal decidua. Together, the placenta and FM ensure materno-fetal interactions, constituting a real interface and complex barrier between the mother and her future child throughout pregnancy. Fetal membranes are composed of two layers: the amnion, which is directly in contact with the AF, and the chorion, which is attached to the maternal decidua [1,2,3,4].

### 1.2. A Multifunctional Barrier: Different Biological Roles of FM during Pregnancy

FM accomplishes several important physiological functions throughout pregnancy until parturition. First, thanks to their elastic resistance strength, they constitute a physical barrier. This is an absolute requirement for maintaining the integrity of the amniotic cavity in the semirigid uterus, while the movements of the AF, which are caused by the development of the fetus, exert permanent pressure in all directions. This resistance and elasticity of the FM are conferred mainly by the extracellular matrix of the amnion [5,6,7]. 

In addition, FMs are also involved in the regulation of AF volume and homeostasis. Indeed, they allow for bidirectional transfers of AF between the amniotic cavity and maternal circulation thanks, for example, to aquaporins [8,9,10]. 

They also constitute a barrier against infections and toxins by releasing some antimicrobial molecules (defensins, lactoferrin, elafin, etc.) [11,12,13]. Furthermore, it has been also described that amniotic tight junctions decrease the spread of microbes in pregnancy, whereas inflammatory mediators can disrupt this barrier, especially in the case of chorioamnionitis [14,15].

**Figure 1:**
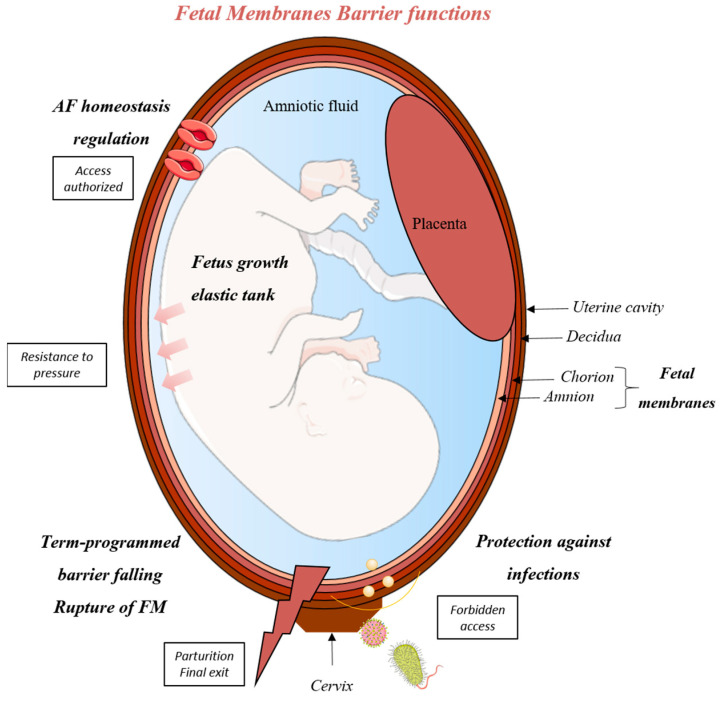
Physiological functions of the FM barrier. FMs are a nine-month organ required for the progress of the pregnancy. Indeed, they exercise different essential functions. First, they constitute an adjustable tank resisting internal and external pressure where the fetus can move and grow. FM also allows the amniotic fluid (AF) to achieve homeostasis by letting ions, water and other compounds pass, notably through aquaporins. On the other hand, they physically and chemically limit the ascension of microbial agents. Finally, they are implicated in parturition by their planned and controlled rupture at term. SMART Servier Medical Art device was used from the creation of the figure.

At the end of this nine-month period, this barrier breaks and the parturition starts, opening the way for the fetus. This phenomenon, called FM rupture, is complex and requires the involvement of many molecular and cellular mechanisms, leading to progressive FM weakening (Figure 1).

## 2. Inflammation or How Does the FM Rupture Occur Right on Time or Too Early?

### 2.1. Happy Ending: Well-Balanced Inflammation for a Well-Prepared Rupture of the FM at Term

Most of the time, FM rupture occurs at the beginning of labor. However, in 10% of term deliveries, it happens before labor, meaning that a rupture is not limited to only being a consequence of labor contractions [16,17]. Most of time, this breakage takes place in a particular zone of the FM facing the cervix called the “zone of altered morphology” (ZAM), which is in opposition to the “zone of intact morphology” (ZIM) (note that sometimes the rupture site is aside the ZAM) [18]. Anatomically, the ZAM presents a thinner structure than the ZIM because of the combination of exacerbated mechanisms in this zone, such as oxidative stress, hormone production, apoptosis, senescence, epithelio-mesenchymal transition, inflammation and extracellular matrix (ECM) degradation [3,18,19,20,21,22]. In addition, these mechanisms are associated with stretching and the appearance of microfractures of the barrier, which become weaker throughout pregnancy [4,23]. 

Recently, more and more studies have particularly focused on the establishment and implication of the inflammation process. Indeed, several results have highlighted a higher inflammation phenomenon in the ZAM. For example, at term, chorion-derived trophoblasts from the ZAM modify their phenotype, inducing maternal immune cell activation. In addition, a transcriptome realized by the Nhan-Chang group revealed a differential expression of inflammatory genes between chorionic ZAM and ZIM [24,25]. More generally, in parallel with this ZAM tissue-specific inflammation, the AF levels of cytokines, such as IL1β, IL6 and TNFα, are increased during labor [26,27]. Furthermore, the release of TNFα induces apoptosis in FM, and IL1β and TNFα lead to downstream chemokine production (IL8, CXCL1, CCL2) associated with the recruitment of leukocytes toward the amnion and chorion [28]. In addition, these pro-inflammatory mediators increase the gelatinase (metalloproteases 2, 9) activity, leading to the degradation of the ECM in the FM (via the nuclear factor-kappa B (NFκB) pathway) [22,29,30,31,32]. 

**Figure 2:**
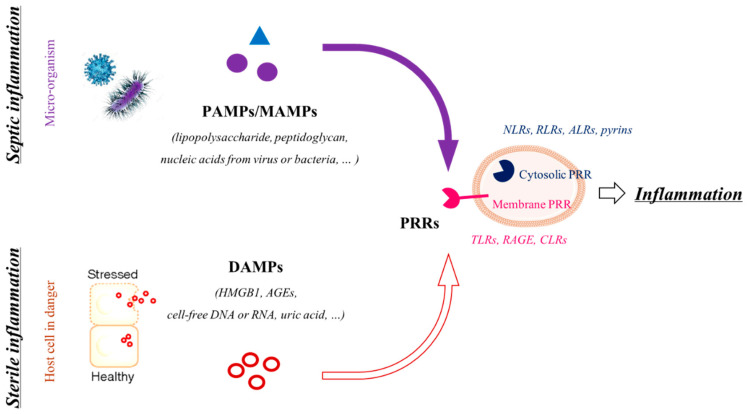
Two types of inflammation. This figure distinguishes the two types of inflammation: (i) the septic one implies the recognition of PAMPs/MAMPs derived from micro-organisms (viruses, bacteria or fungi); (ii) the sterile one is triggered by the recognition of DAMPs by the members of both PRRs (cytosolic or membrane-addressed). Both the recognition of PAMPs/MAMPs and DAMPs lead to a pro-inflammatory response.

Thus, inflammation appears to be central in the physiological weakening of FM, and this response is set up sequentially during pregnancy. Furthermore, this end-of-pregnancy inflammation is mainly microbial free and, thus, is called sterile and results from the recognition of some specific molecules, the alarmins or DAMPs (damage-associated molecular patterns). Several types of DAMPs (high-mobility group box 1 (HMGB1), uric acid, cell-free DNA, etc.) are released by senescent FM cells the closer the pregnancy comes to term; they spread throughout adjacent tissues as a consequence of cell apoptosis or are encapsulated in small extracellular vesicles called exosomes, amplifying both inflammation and senescence. In fact, for example, a gradual increase in the maternal plasma exosomes containing pro-inflammatory agents was discovered during mouse gestation [33]. They have been reported in gestational tissues during labor and described as responsible for the activation of various pro-inflammatory pathways, such as the p38 MAPK or NFκB pathways [34,35]. To act as “inflammation promoters,” DAMPs are recognized by the receptors of innate immunity: the PRRs (pattern recognition receptors). It is worth noting that PAMPs/MAMPs (pathogen-associated molecular patterns/micro-organism associated molecular patterns) are recognized by the same receptors in the case of a microbial response. Two classes of PRRs have been described: the membrane PRRs and cytosolic PRRs, allowing for a recognition of a danger signal outside and inside the target cells, respectively. Among the membrane-bound PRRs are the CLRs (C-type Lectin receptors), TLRs (toll-like receptors) and, more recently, RAGE (receptor for advanced glycation end products). Alternatively, NLRs (nucleotide-binding and oligomerization domain (NOD)-like receptors), RLRs (retinoic acid-inducible gene-I (RIG-I)-like receptors), ALRs (absent-in-melanoma (AIM)-like receptors) and the pyrins are cytosolic PRRs [36,37] (Figure 2). 

As mentioned above, inflammation is an essential component of the physiological process of FM weakening. Nevertheless, as Paracelsus said, “The dose makes the poison” (or should we say, DAMPs make the way out). Indeed, even if this physiological well-balanced inflammation leads to a healthy birth right on time, when it is overly exacerbated or not well-regulated, it can result in preterm prelabor rupture of the FM and an unfortunate preterm birth.

### 2.2. Preterm Prelabor Rupture of Fetal Membranes (pPROM)

#### 2.2.1. Definition and Consequences of the pPROM

In obstetrics, pPROM is defined as a rupture of the FM before the onset of labor. It can happen at any gestational age. pPROM affects 2–4% of singleton pregnancies and 7–20% of twin pregnancies [16]. This complication is responsible for one-third of preterm births (PTB) and is associated with an elevation of perinatal and maternal mortality [16,38,39]. Indeed, pPROM increases the risk of placental abruption, postpartum endometritis and chorioamnionitis [40,41]. Such early inflammation of the placenta and FM can be caused by the rise of genital germs or by contamination following invasive medical procedures. The incidence of chorioamnionitis is directly related to the duration of the latency period between FM rupture and childbirth [42,43]. Finally, pPROM leads to a decrease in AF volume, which is potentially associated with umbilical cord compressions or prolapses. pPROM can also lead to neonatal infection, though its main outcome is PTB, whose consequences vary depending on the gestational age (developmental disorders of lungs, intestines, sensorial organs, nervous system, etc.) [44].

#### 2.2.2. Pathophysiology of pPROM and Inflammation

As previously described, membrane rupture results from a complex and multifactorial chain, leading to a progressive weakening of the FM. pPROM results from this same chain but in an earlier and accelerated way. We can imagine that while the FM normally takes the time on the slow lane, those of mothers undergoing a pPROM burn the speed limits, arriving too early at the final destination: their decay. This pathology is described as being the consequence of a myriad of risk factors whose origin can be maternal (smoking, drug consumption, ethnic polymorphisms, nutrition), feto-utero-placental (multiple pregnancies, infection, premature labor), environmental (pollutants, lead, etc.) or iatrogenic (amniocentesis, etc.) [45,46,47]. 

Indeed, sterile or septic inflammation is considered essential in the pathogenesis of pPROM. Several inflammatory markers have been found to increase in AF, maternal blood, the placenta and the FM in cases of pPROM [48,49,50,51,52,53]. These higher levels of pro-inflammatory cytokines have been correlated with the recruitment of leukocytes (neutrophils, monocytes, macrophages, T cells) within the AF of pPROM patients, especially those with a positive bacterial culture [49,54]. Conversely, higher levels of anti-inflammatory cytokines (IL1RA) are associated with a longer latency (>7 days) to delivery in women with pPROM [55]. Thus, many interleukins have been studied as the potential biomarkers of pPROM, such as IL6 [56,57,58]. If it is well known that an infectious stimulation provokes an inflammatory response, in particular at the chorion level (first target of vaginal pathogens); here, some teams have started to study sterile inflammation (potentially caused by smoking, nutritional deficiencies, pollutants, etc.) in the context of pPROM [59,60]. Indeed, its role is not negligible. A study showed that 58% of patients with sterile intra-amniotic inflammation have histological chorioamnionitis features (infiltration of polynuclears at the level of the fetal membranes) [61]. Moreover, higher concentrations of alarmins such as HMGB1, S100β, heat shock protein 70, cell-free DNA and S100 proteins have been found within AF, maternal blood or even gestational tissues (placenta, FM) in cases of pPROM or chorioamnionitis [62,63,64,65,66,67,68,69]; these increased amounts of DAMPs, which can lead to an inflammatory stimulation by interacting with PRRs, might be the consequence of oxidative stress or senescence [70,71]. Finally, a study revealed that the in vivo inhibition (in a murine model) of the inflammatory pathway NFκB causes delays in lipopolysaccharide (LPS)-induced PTB, clearly showing the importance of inflammation in parturition [72].

In the following parts of this review, special attention will be paid to the importance of the RAGE membrane receptor and to an intracytoplasmic receptor family that can form an inflammatory platform: the inflammasome. 

## 3. The Receptor for Advanced Glycation End Products (RAGE)

### 3.1. RAGE Protein Structure

Discovered in 1992 by Michael Neeper, the RAGE protein (molecular weight of about 46 kDa) is a membrane receptor belonging to the immunoglobulin superfamily and induces downstream different intracellular signals. Globally, it is expressed at low levels in all human tissue, except in the lungs, where its expression is more important [73,74]. Structurally, the full-length RAGE (fl-RAGE) is composed of three domains: an extracellular domain (itself subdivided into three parts involved in ligand recognition: V, C1 and C2), a transmembrane domain and a cytoplasmic portion required for signal transduction by its interaction with adaptor proteins (Myeloid differentiation primary response 88 (MyD88), Diaphanous-1 (Dia-1) and the Toll-interleukin 1 Receptor (TIR) domain containing adaptor protein (TIRAP)) [75,76,77,78]. 

**Figure 3:**
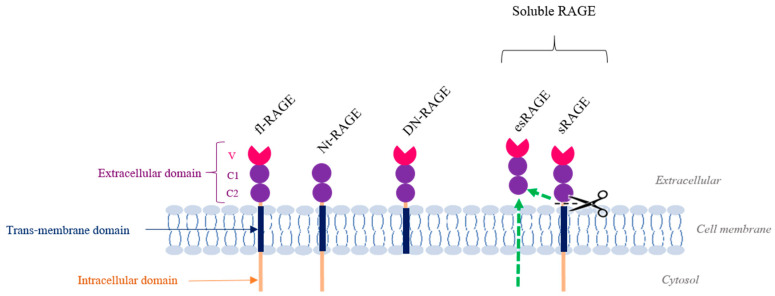
RAGE and its isoforms. This figure illustrates the different protein isoforms of RAGE. Only the full-length (fl-RAGE) version can exercise a complete signal transduction by first interacting with ligands with its extracellular domain (V: variable; C: constant) similar to immunoglobulins and then recruiting protein adaptors using its intracellular domain. Other isoforms, especially soluble ones (esRAGE, directly translated from a transcript and sRAGE, resulting from an enzymatic cleavage of fl-RAGE), are implicated in the negative feedback of RAGE activity by competition. Finally, Nt-RAGE and DN-RAGE have also been briefly described in the literature.

RAGE also exists in truncated forms. Two of them are soluble: sRAGE (soluble RAGE), which is derived from a proteolytic cleavage of fl-RAGE by specific metalloproteases, and esRAGE (endogenous secretory RAGE), which is derived from one specific splicing transcript [79,80]. Functionally, these two forms, which lack the transmembrane and intracellular domains, act as inhibitors of signal transduction by capturing RAGE ligands in the extracellular environment before their recognition by the functional fl-RAGE. There is also an isoform named DN-RAGE (dominant negative), which is addressed to the membrane but does not have an intracellular domain. Finally, Nt-RAGE (N-terminal truncated), resulting from alternative splicing, is V-domain lacking, so it cannot bind to the usual ligands (Figure 3) [74].

### 3.2. RAGE, Ligands and Signaling

Thanks to its multiple extracellular domains (V, C1, C2), RAGE can interact with a considerable panel of ligands, mainly endogenous ligands that are accumulated in tissues during aging, inflammation and other stresses: the DAMPs are also called alarmins [81,82]. Among these ligands, RAGE can interact with the HMGB1, an S100 protein family, advanced glycation end-products (AGEs) derived from the Maillard reaction of glycation, cell-free DNA and RNA, uric acid... This diversity of ligands, which is associated with other factors such as cell type, ligand or receptor concentration at the membrane or type of adaptors, allows RAGE to induce numerous signaling pathways, leading to various cell responses. For example, RAGE has been described in cell migration and proliferation but also in apoptosis [83,84]. A recent study also demonstrated that RAGE can induce autophagy [85]. Finally, one of the main roles of RAGE is its ability to initiate an inflammatory response [75].

### 3.3. RAGE and Inflammation

**Figure 4:**
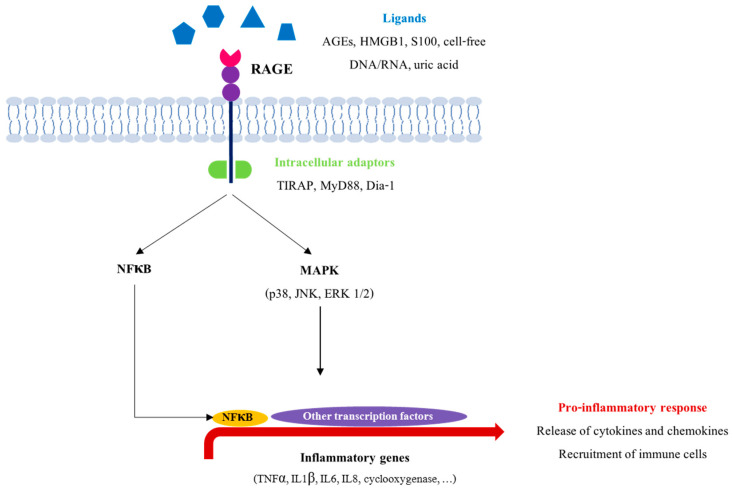
RAGE signaling pathway. Following the binding of its ligands, RAGE can induce different proinflammatory pathways, implicating some adaptor intracellular proteins (TIRAP, MyD88, Dia-1). This activation results in the induction of proinflammatory gene transcription (TNFα, cyclooxygenase 2, IL1β, IL6, IL8), thus maintaining the inflammation phenomenon.

In the literature, RAGE exhibits high levels of expression in the inflammatory lesions associated with many types of pathologies. On the other hand, blocking RAGE delays the inflammatory response [86]. For example, RAGE knockout mice are protected against inflammatory disorders (sepsis, smoking-induced lung inflammation, etc.) [87,88]. In addition, RAGE is expressed in many immune cells, and an accumulation of its ligands is often observed at inflammation sites [89,90,91]. The stimulation of RAGE initiates various pro-inflammatory pathways. For example, the NFκB pathway was the first signal described as activated by AGEs [92]. This transcription factor regulates the expression of several genes, including pro-inflammatory mediators (TNFα, IL1β, IL6, cyclooxygenase 2, etc.) and RAGE itself, amplifying chronic inflammation [93]. Activation of RAGE by its ligands can also lead to the induction of MAP kinase signaling cascades, such as ERK, JNK or p38-MAPK (Figure 4) [94,95]. 

### 3.4. RAGE and Fetal Membranes Rupture

Despite the well-described role of RAGE in inflammatory pathologies, only a few studies have investigated its potential implication in FM inflammation and their subsequent rupture. Romero et al. demonstrated higher AF levels of sRAGE and esRAGE in the presence of intra-amniotic inflammation and infection [96]. Nevertheless, under the same conditions, another study demonstrated that these levels are not impacted by the presence of intra-amniotic inflammation/infection, instead revealing the expression of RAGE within the amnion and chorion [97]. Later, other studies reported an increase in plasmatic esRAGE levels in pPROM but also in placental RAGE levels [63,98]. However, more recently, in 2020, another study found no significant difference in plasmatic sRAGE levels between pPROM and term rupture [99].

Concerning its ligands, DAMPs are found in AF and gestational tissues and are often dysregulated in cases of pregnancy pathologies such as pPROM. For example, overexpression of HMGB1 in the sera and placentas of mothers undergoing pPROM has been described [63,100]. In addition, a preliminary study supported the idea of using AGEs as an early (first trimester) serum marker of preterm labor and pPROM [101]. Furthermore, a direct link was demonstrated between the risk factors of pPROM (e.g., smoking) and the release or production of alarmins; for example, it has been well described that smoking induces alarmin release [102].

Nevertheless, a recent study clarified the implication of RAGE in FM weakening because of sterile inflammation [103]. Indeed, the ontogeny of the RAGE axis in FM obtained from nonpathological deliveries at term by cesarean produced the first data about the expression of its adaptors in these tissues, which is essential to RAGE activity. It was elucidated that the FM expressed RAGE and HMGB1 but also two intracellular adaptors (Dia-1 and MyD88) during the three trimesters of human pregnancy. Furthermore, the RAGE protein was identified in the amniotic epithelium, the first cells in contact with AF alarmins and in the chorion too [103]. A comparison of the RAGE actors’ expressions between the two layers of FM, but also between the ZAM (rupture zone, the weaker) and ZIM, revealed that RAGE and HMGB1 expression is significantly higher in the amnion compared with chorion. Even better, an overexpression of RAGE was found in the ZAM compared with the ZIM for the amnion (layer considered the stronger in FM) [103]. This suggests an overactivation of RAGE in the amnion around the zone of rupture, which is probably involved in its weakening. Romero et al. described an HMGB1-associated sterile inflammation on the fetal side of the FM [66]. Moreover, the overexpression of Dia-1 was revealed in amnion, whereas MyD88 was found overexpressed in chorion [103]. These differences could be considered a clue for layer-specific signaling, here depending on the recruited adaptor. Such consideration is supported by the fact that it is already well known that MyD88 is shared with TLR2/4 and TLR4 is highly expressed in the FM as well, opening the way to study the hypothesis of cooperation between RAGE and TLR4 in FM weakening [104].

In parallel, AGEs and HMGB1, which are both alarmins found to be increased in the case of pPROM, can induce the sterile inflammation of FM. Indeed, some studies have demonstrated that they induce cytokine release (IL8, IL6, TNFα, IL1β) in the FM without a distinction between amnion and chorion [35,105]. Later, these first data were complemented by revealing that amnion and choriodecidua did not respond in the same way to the stimuli from alarmins. Both layers respond to alarmins by the release of TNFα and IL1β, but only the amnion produced IL6 following the treatment. Above all, RAGE was found to be required in the FM layers to induce a pro-inflammatory signal after AGEs or HMGB1 exposure. Indeed, a total abortion of HMGB1-induced inflammation by the RAGE inhibitor was observed, but only partially decreased AGEs-induced inflammation [103]. These differences remind us of the complexity of RAGE activity, depending on the ligand type and ligand concentration. To conclude, recent data support the importance of RAGE engagement in FM sterile inflammation because of alarmins. Obviously, further studies are required to describe this RAGE activation in the FM, the adaptor recruitment and specificity of actions, here investigating downstream cell pathways (MAPK, NFκB?), the type of cell responding in the tissue and possible cooperation with other inflammation actors, such as the inflammasome platforms described below.

## 4. Inflammasomes

### 4.1. What Are Inflammasomes?

The first inflammasome was discovered in 2002 by the biochemist Jürg Tschopp [106]. This molecular platform can recognize, as a PRR, the DAMPs and PAMPs and induce inflammatory processes, especially pyroptosis, which is a recently described inflammation-specific cell death [107,108]. In fact, an inflammasome is an intracellular complex composed of three actors: a sensor, an effector (the pro-caspase 1) and an adaptor between the two previous parts. These inflammasomes are formed by cytosolic PRRs, such as NLRs; however, membrane PRR activation can stimulate the downstream formation of inflammasomes [109,110]. In this review, we focus on one subfamily of NLRs, the NLRPs (NLR and Pyrin domain containing), which includes 14 members: NLRP1 to NLRP14. 

#### 4.1.1. The NLRP Inflammasome: The Trio NLRP/ASC/pro-Caspase 1 

**Figure 5:**
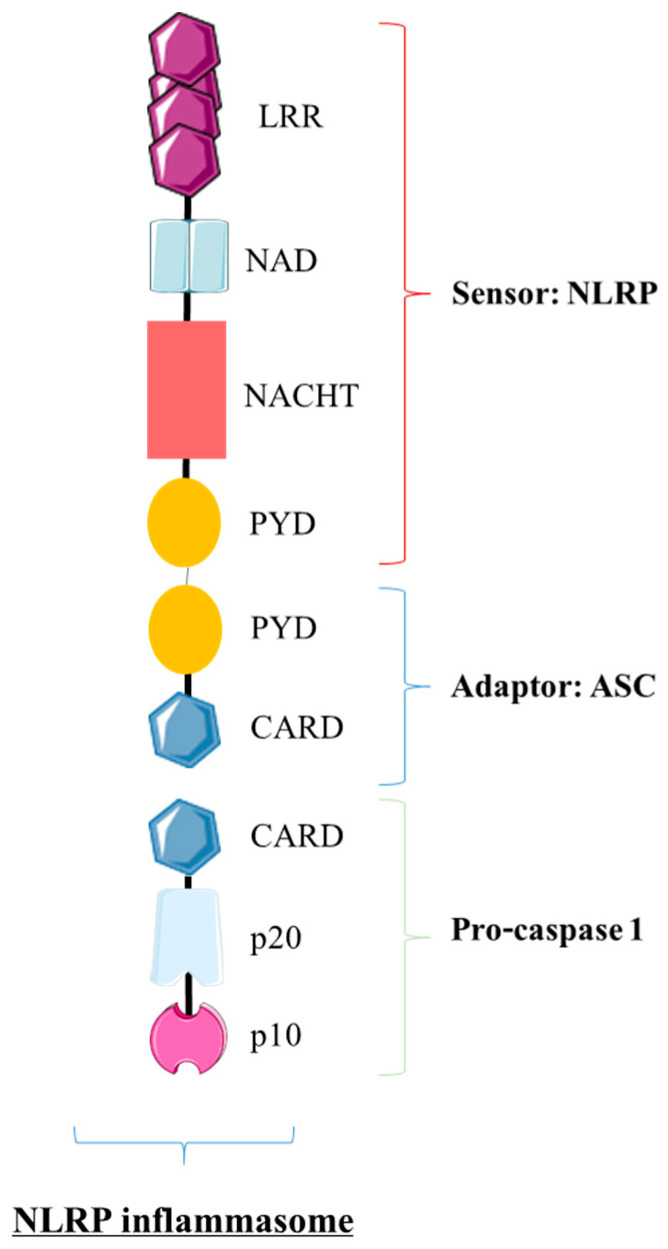
NLRP inflammasome structure. A NLRP inflammasome is composed of three major elements. First, there is a sensor, the receptor NLRP, which itself is generally composed of different domains: PYD (Pyrin domain), NACHT (acronym for NAIP, CIITA, HET-E, TP1), NAD (NACHT-associated domain) and LRR (Leucine-rich repeats). This LRR domain allows for an interaction with the adaptor ASC by the PYD domains. Then, ASC can recruit pro-caspase 1 thanks to the CARD (caspase recruitment domain) of each actor. In addition, pro-caspase 1 also contains a central p20 domain (including the catalytic site) and a p10 domain.

For this group, the inflammasome includes one NLRP as a sensor linked to the procaspase 1, here thanks to the ASC adaptor (apoptosis-associated speck-like protein containing a caspase recruitment domain (CARD)). More precisely, a NLRP (except for NLRP1 and 10) is generally composed of these domains: a pyrin domain (PYD) followed by a central domain NACHT (NAIP, CIITA, HET-E, TP1 proteins) involved in oligomerization; a domain associated with NACHT (NAD); and, finally, a carboxy terminal domain made of Leucine-rich repeats (LRR) [111,112,113]. During the assembly of the inflammasome, the NLRP interacts with the adaptor ASC because of the PYD domain of each actor. the ASC also contains a CARD domain that binds the CARD domain of pro-caspase 1 [114,115]. Caspases are divided into two classes: pro-apoptotic and pro-inflammatory caspases. Caspase 1 belongs to the second one and is included in the inflammasome as a pro-caspase 1 protein composed of three domains: CARD, p20 and p10 (Figure 5). Following such an association, inflammasomes assemble together, leading to the formation of a specific structure called specks [116,117,118]. 

#### 4.1.2. NLRP Inflammasome Activation

**Figure 6:**
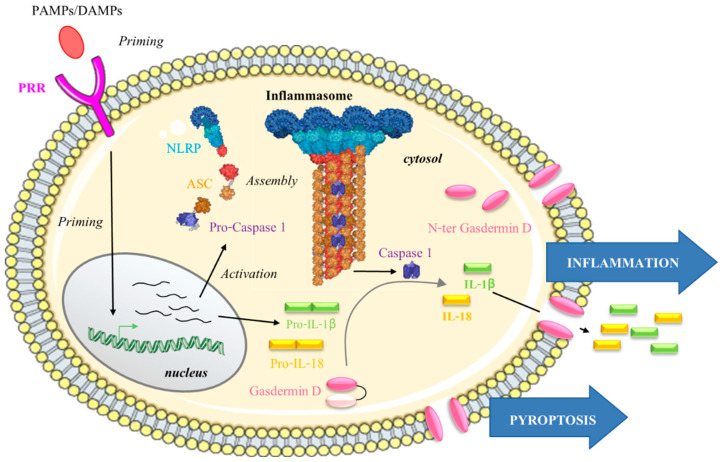
NLRP inflammasome activation. Following the priming step (transcriptional and/or not), the activated NLRP can form the inflammasome with ASC and pro-caspase 1. This functional NLRP inflammasome leads to the autolytic cleavage of pro-caspase 1 into caspase-1, which is the active enzyme. Downstream, caspase-1 activates the interleukins IL18 and IL1β but also releases the N-terminal fragment of the gasdermin D from the inhibitory C-terminal fragment. Finally, a formation of pores by the N-terminal gasdermin D at the plasma membrane induces pyroptosis and propagation of the inflammatory signal [117,124,126].

Based on studies about NLRP3, two activation pathways have been described. To form a functional NLRP inflammasome, the canonical pathway requires two steps: (i) a priming pro-inflammatory actor recruitment or transcription, here following DAMP/PAMP recognition by a PRR, and (ii) induction of the assembly of an active multiprotein complex. Priming can allow for the transcription of NLRPs, ASC, pro-caspase 1 and pro-interleukins 18 and 1β. Furthermore, non-transcriptional priming can occur and modulate the post-translational modifications required for inflammasome availability [119,120,121,122]. Once the inflammasome is assembled, the pro-caspase 1 is maturated in caspase 1, which cleaves pro-IL18 and pro-IL1β in active forms (triggering inflammation), and a protein named gasdermin D leads to the generation of an active N-terminal portion. Such final active portions assemble to form pores at the cell membrane, inducing pyroptosis (i.e., cell lysis) and inflammation through the consequent release of intracellular contents, including IL18, IL1β and inflammasomes specks, provoking an amplification of the inflammatory signal and immune cell recruitment [123,124,125] (Figure 6). 

Thus, regarding their inflammatory action, NLRP inflammasomes are already implicated in different pathogeneses, as is the case more often in pathological pregnancies [127,128,129,130,131,132,133].

### 4.2. NLRP Inflammasomes, FM Diseases and pPROM

Studies on the pathological implication of NLRP inflammasomes (especially NLRP3) have focused on preeclampsia, an obstetrical disease where the uric acid levels are particularly increased in the blood [127,134,135]. Indeed, uric acid has been found to provoke an ASC-dependent activation of caspase 1 and IL1β release in vitro, which is associated with an overexpression of NLRP3 in human first trimester trophoblasts [136,137]. 

Furthermore, in the case of chorioamnionitis, a risk factor of pPROM, FM exhibits higher expression of inflammasome actors. In addition, one study demonstrated that AF from mothers with intra-amniotic inflammation (IAI) or infection contains more important ASC levels [138,139]. These data implicate NLRP inflammasomes in septic or sterile inflammation occurring during chorioamnionitis, a disease affecting FM barrier integrity.

In preterm labor, the concentration of caspase-1 in the AF is higher if labor is associated with infection and/or IAI when compared with a situation of preterm labor without infection and/or inflammation [140]. Gasdermin D has also been detected in the AF and FM in preterm labor, suggesting that pyroptosis may be involved in preterm labor and PTB [141]. Therefore, preterm labor seems to involve inflammasomes, especially in the case of infection or IAI [142]. In addition, Motomura et al. demonstrated in vivo that IL1α induces PTB through the NLRP3 inflammasome [143]. This NLRP3 implication is confirmed by Faro’s team, who induced PTB in an animal model by LPS-induced IAI, a phenomenon that was reduced by the use of a specific inhibitor of NLRP3 [144].

Finally, one study shows that in pPROM, the expression of transcripts and proteins related to the inflammasome signaling pathway (Nucleotide-binding oligomerization domain-containing protein 1 (NOD1) and NFκB) are increased in the placenta and FM [145]. In addition, Theis et al. examined the AF in mothers undergoing pPROM. The results revealed a positive correlation between microbial burden and ASC and IL6 concentrations [146]. Recently, another study highlighted higher expression levels of NLRP3, NLRP1 inflammasomes, IL18 and IL1β into the placenta and FM in the case of pPROM [147]. To conclude, all of these data demonstrate a clear link between NLRP inflammasomes and pPROM.

### 4.3. Focus on NLRP7 

As detailed before, the NLRP family is composed of 14 members. Personal unpublished data identified the NLRP members expressed in the human amnion and chorion (collected at term) but also the inflammasome actors ASC and caspase-1 (Table 1).

In the obstetrical sphere, studies have been more focused on NLRP3, sometimes NLRP1, but some new data have recently emerged on NLRP7. In a more functional view, the NLRP7 inflammasome has been described as being involved in the activation of caspase-1 and IL1β release following infection of THP-1 cells (monocytes cell line) by *Mycobacterium bovis*. This activation of the NLRP7 inflammasome induced cell death, which the authors suggested to be pyroptosis [148]. Another team focused on the post-translational modifications regulating NLRP7. Indeed, the NLRP7 protein is constitutively ubiquitinated and recruited to the endolysosome to be degraded. Upon stimulation by a pro-inflammatory ligand, the STAMBP (Signal transducing adaptor molecule binding protein) enzyme deubiquitinates NLRP7, preventing its degradation and allowing the formation of an active inflammasome that activates IL1β [149]. Finally, Abi Nahed et al. recently demonstrated that the activation of the NLRP7 inflammasome in a trophoblastic cell line induced the secretion of IL1β but not IL18 [150]. Numerous studies have described the involvement of the NLRP7 inflammasome in some obstetrical complications, such as the recurrent hydatidiform mole (a disease characterized by an excessive trophoblastic proliferation without embryonic development), especially those related to mutations of NLRP7 [151,152,153,154,155]. In addition, the NLRP7 inflammasome is highly expressed by placental trophoblast cells and is deregulated in fetal growth restrictions, a disorder importantly related to abnormal placental development [150]. In addition, Tsai et al. demonstrated that NLRP7 relates to decidualization and macrophage differentiation, supporting their hypothesis of a multitasking gene implicated in endometrial hemostasis and reproductive success [156]. 

To conclude, NLRP7 seems important for inflammation response and pregnancy success. This is why, in the last part of this review, special emphasis will be placed on the emerging data about its implication in FM inflammation establishment.

### 4.4. Does NLRP7 Play a Role in FM Rupture?

The presence of the three NLRP7 inflammasome actors (ASC, caspase 1 and NLRP7) has been characterized in the amnion and chorion but also in primary amniotic epithelial cells (AECs) that were collected from the FM of women with term deliveries. It suggests that this NLRP7 inflammasome can assemble in the FM at all stages of pregnancy [157]. According to the literature, only one ligand is known and has been used to specifically activate the NLRP7 inflammasome: fibroblast stimulating lipopeptide (FSL-1) [158]. FSL-1 is a synthetic lipopeptide derived from the lipoprotein of *Mycoplasma salivarium*, representing the N-terminal portion of the LP-44 lipoprotein of this mycoplasma [159]. It is strongly similar to another lipopeptide, MALP-2 (Macrophage-activating lipopeptide-2), which is derived from the lipoprotein of *Mycoplasma fermentans* [160]. Numerous studies have used FSL-1 on FM, amnion cells or myometrial cells [22,161,162,163,164,165,166,167,168,169,170]; they allowed for the characterization of the signaling pathway induced by FSL-1, and they demonstrated an induction of the synthesis of pro-inflammatory mediators. The NLRP7 inflammasome, though specifically activated by this ligand, has been studied in the FM context in only one study [157].

Thus, Lavergne et al. demonstrated that AECs can form a functional NLRP7 inflammasome. More interestingly, the quantification of the expression of these three actors in the FM revealed an overexpression of ASC (in both layers of FM) at term with labor compared with term FM without labor [157]. This observation confirms the study of Gomez-Lopez et al.*,* who described an increase of ASC/Caspase-1 complexes in the FM with labor [138]. No difference was found for NLRP7, whereas Romero et al. demonstrated an overexpression of NLRP3 protein in the FM with labor [171].

Furthermore, as previously mentioned, NLRP7 is activated by FSL-1, which is derived from *M. salivarium*, and similar to a lipoprotein from *M. fermentans*. Mycoplasmas have been studied extensively for their involvement in pPROM pathogenesis. Numerous mycoplasmas have been identified in the genital tract (*M. genitalium*, *M. hominis*, *M. fermentans*, *M. penetrans*, *Ureaplasma* spp., etc.), and some of them have been found to be related to pPROM or chorioamnionitis [172,173,174,175,176,177,178,179]. Moreover, Lavergne et al. found the presence of *M. fermentans* and *M. salivarium* in healthy human FM after vaginal deliveries but also after cesarean deliveries, proving that bacteria detection was not because of contamination after crossing the genital tract. These observations imply a genital tract ascension, even in healthy carriers. However, it can be supposed that overproliferation might lead to a pathological outcome, such as for the other mycoplasmas described in the literature. 

In the aforementioned study, the *M. salivarium*-derived lipopeptide FSL-1 not only induced the expression of NLRP7 and caspase-1 in the AECs but also allowed the colocalization of ASC and NLRP7 around the nuclei, proving inflammasome assembly. In parallel, FSL-1 leads to an increase of IL1β transcription but not IL18, bringing to the fore the study of Abi Nahed et al. on trophoblasts, where only an induction of IL1β was found in response to NLRP7 inflammasome [150]. In addition, FSL-1 induces the cleavage of gasdermin D into its pyroptotic fragment. These experiments prove the importance of the NLRP family members, which are directly linked to cell death, inflammation and DAMP release, all of which are mechanisms involved in FM rupture. 

## 5. Conclusion

This review has summarized recent data about the implication of inflammation in FM weakening. Furthermore, two new actors seem particularly interesting to explore for their inflammatory ability: RAGE and NLRP7. Both might be overactivated in risk contexts (smoking, germs rising in excess, chorioamnionitis) and be involved in pPROM pathogenesis. More studies are required to know in detail the implication of RAGE and its adaptors, but also of NLRP inflammasome pathophysiological weakening of FM to elucidate the link between the materno-fetal exposome, the PAMP/DAMPome and pPROM.

## Figures and Tables

**Table 1:** Expression of the NRLP family in human FM. The mRNA expression of the 14 NLRPs, ASC and caspase-1 was investigated by RT-qPCR in terms of human amnion and chorion. “+” indicates the presence of the transcripts, and “Ø” indicates the absence of RNA expression.

	NLRP														ASC	Caspase-1
	1	2	3	4	5	6	7	8	9	10	11	12	12	14		
Term Amnion	+	+	+	Ø	Ø	+	+	Ø	+	+	Ø	Ø	Ø	+	+	+
Term Chorion	+	+	+	Ø	Ø	+	+	Ø	+	+	Ø	+	Ø	+	+	+

## Data Availability

Not applicable.
